# Endemic *Paragonimus kellicotti* infections in animals and humans in USA and Canada: Review and personal perspective

**DOI:** 10.1016/j.fawpar.2022.e00184

**Published:** 2022-12-09

**Authors:** J.P. Dubey

**Affiliations:** United States Department of Agriculture, Agricultural Research Service, Beltsville Agricultural Research Center, Animal Parasitic Diseases Laboratory, Building 1001, Beltsville, MD 20705-2350, USA

**Keywords:** *Paragonimus kellicotti*, Life cycle, Diagnosis, Animals, Humans, Epidemiology, Treatment

## Abstract

Infections with the lung fluke, *Paragonimus kellicotti,* have been diagnosed in a variety of domestic and wild animals and humans in USA and Canada. Although there are many species of *Paragonimus* in other parts of the world; *P. kellicotti* is the only species definitively diagnosed in USA and Canada. Fresh water snails (several species) and crayfish (mainly *Orconectes* spp.) are its intermediate hosts. Humans and animals become infected with *P. kellicotti* only by ingesting metacercariae encysted in the heart of crayfish. After ingestion, the fluke penetrates intestinal wall, enters peritoneal cavity, and reaches pleural cavity by direct penetration of diaphragm, 2–3 weeks post inoculation (p.i.). Young flukes penetrate lungs and become encysted in pulmonary tissue, often in pairs. Time to maturity is around 4–7 weeks p.i. Eggs are coughed up, swallowed, and are excreted in feces. Although the parasite has been known for more than a century, there has been an upsurge of human infections in the USA. Here, I review *P. kellicotti* infections in naturally infected hosts. Pathogenesis, diagnosis, and treatment in parasite-free cats and dogs experimentally infected *P. kellicotti* are reviewed to shed light on the pathogenesis of human paragonimiasis. Problems and challenges facing diagnosis of paragonimiasis, especially non-pulmonary infections, are discussed. Fluke stages are deposited in Smithsonian Museum.

## Highlights

• *Paragonimus kellicotti* is an emerging (or better diagnosed) zoonosis in the USA.
• Updated review of biology of *P. kellicotti* infections in animals and humans.
• Eating uncooked infected crayfish (not farm raised) main risk factor for *P. kellicotti* infections in humans.
• *Paragonimus kellicotti* specimens deposited in Smithsonian Museum.

## Graphical abstract

**Figure:**
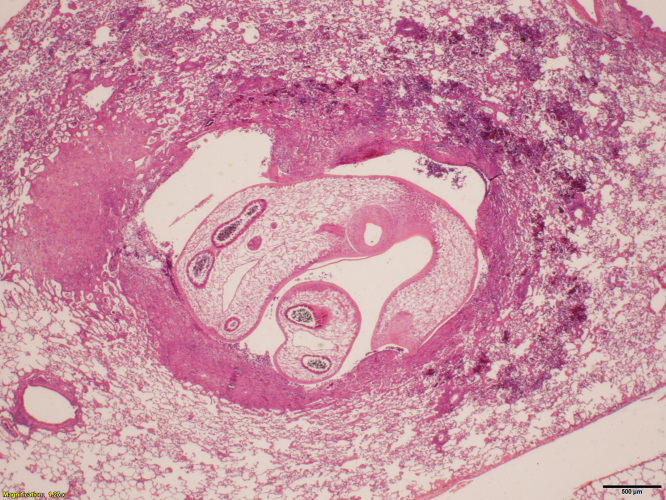

## 1 Introduction

Paragonimiasis is parasitic infection of animals and humans recognized for more than a century and there are several reviews on this topic (Yokogawa, 1969; Procop, 2000; Diaz, 2013; Fischer and Weil, 2015; Yoshida et al., 2019; Blair, 2022; Le et al., 2022). There are many species of *Paragonimus* (Blair, 2022). The disease in the Far East caused mainly by *Paragonimus westermanni* is well characterized (Procop, 2000). The recent reports of human paragonimiasis in USA caused by *Paragonimus kellicotti* infections in North America have aroused the interest of public health authorities and clinicians (reviewed by Coogle et al., 2022). *Paragonimus kellicotti* is the only species of *Paragonimus* established as endemic in USA.

Compared to human infections, much more is known concerning *P. kellicotti* infections in domestic and wild animals in North America. Adopting a One Health approach to aid zoonotic risk assessment and treatment of human infections, I hereby review the body of knowledge derived from natural and experimental studies in animals with the interest that the information might be useful for pathogenesis of infection in humans. This paper exclusively discusses *P. kellicotti* infections in the USA and Canada.

## 2 History and personal prospective

The lung fluke later named *Paragonimus kellicotti* was first discovered in a dog by Kellicott (1894). Because this reference is not easily accessible, I summarize those findings. Professor David Simmons Kellicott was chair of Zoology and Comparative Anatomy at the Ohio State University; then, the chair oversaw all veterinary and human biology (Bleile, 1898). In March 1893, an apparently asymptomatic shepherd dog was used for teaching anatomy to veterinary students. After euthanasia, the dog was bled out, “its blood vessels washed out and injected with plaster” (Kellicott, 1894). The entire surface of pleura and lungs had tumor-like lesions, filled with pus and debris. Twenty flukes were recovered from cutting open the lung nodules. Masses of fluke eggs covered lungs and pleura. He partly described morphology of adult flukes and eggs but did not name the parasite.

At about the same time, flukes were found in the lung of a cat from Michigan (Ward, 1894). In June 1893, a piece of affected lung from a cat in Ann Arbor, Michigan was sent to Professor Henry B. Ward at the University of Nebraska. (For readers not familiar with the history of parasitology in the USA, Dr. Ward founded the Journal of Parasitology and remains a giant of the field). Ward was unfamiliar with the morphology of the lung fluke; he thought the parasite was *Distoma westermanni*. A detailed study of the fluke in cats was hampered because the specimen had been stored in 50% alcohol for an unknown time, and the worms were partly macerated. Although Ward (1894) did not specify the type of cat, presumably it was a domestic cat because Ward considered the possibility that the cat might have been an imported pet; in his review on paragonimiasis, Procop (2000) wrongly stated it was a tiger.

Apparently, Ward (1894) was unaware of the paper by Kellicott. Kellicott subsequently sent flukes from the dog to Ward who named it *P. kellicotti* (Ward, 1908). Subsequently, Ward and his doctoral student Hirsch (Ward and Hirsch, 1915) made a detailed morphologic study of *P. kellicotti* from the dog collected by Kellicott (1894), flukes from the cat from Michigan (Ward, 1894), domestic cats from Wisconsin, and flukes collected from pigs slaughtered at Cincinnati, Ohio (Stiles and Hassall, 1900 discussed later in this review); all were thought to be *P. kellicotti.* Ward and Hirsch (1915) reviewed other reports of *P. kellicotti* infections in cats in USA including the report of Ward and Hirsch (1915). (For citations of these old papers see Ward and Hirsch, 1915). Ward and Hirsch (1915) distinguished *P. kellicotti* from other *Paragonimus* species.

The life cycle of *P. kellicotti* and its morphology remained unknown until the 1930s. After three brief but important reports (Wallace, 1931; Ameel, 1931, Ameel, 1932a), its life cycle was reported in a landmark study by Ameel (1934). He provided detailed information on its morphology and life cycle. Fresh water snails and crayfish were its intermediate hosts (Ameel, 1934) (Table 1). La Rue and Ameel (1937) provided detailed data on the distribution of the fluke in its definitive and intermediate hosts in the Americas; therefore, I have reviewed papers published after 1937. Additional reports (Ameel, 1932b; Ameel et al., 1951; Chen, 1937; Sogandares-Bernal, 1966; Lumsden and Sogandares-Bernal, 1970; Ishii, 1966; Weina and England, 1990; Fischer et al., 2011; Lloyd et al., 2015) contributed to the life cycle of *P. kellicotti*.

**Table 1:** Summary of life cycle stages of *Paragonimus kellicotti.*

Stage/character	Observation	Comment	Main references
Definitive hosts-natural	Mink (*Mustela vison*), domestic and wild cats (*Felis catus*), bob cat (*Lynx rufus*), dog (*Canis domesticus*), coyote (*Canis latrans*), raccoon (*Procyon lotor*), skunk (*Mephitis mephitis*), red fox (*Vulpes vulpes*), weasel (*Mustela* sp.), humans (*Homo sapiens*)	Mink is considered the main natural host	Reviewed by Procop (2000); Diaz (2013); Coogle et al. (2022)
Definitive host-experimental	Musk rat (*Ondatra zibethicus*), albino rat (*Rattus norvegicus*), Syrian hamster (*Mesocricetus auratus*), Mongolian gerbil (*Meriones unguiculatus*)	Although fluke can mature in these rodents, they are unsuitable for life cycle studies	Ameel (1934); Weina and Burns (1992); Fischer et al. (2011)
First intermediate host	Fresh water snail (Families Hydrobiidae, Pleuroceridae) as natural host. Experimentally, *Pomatiopsis lapidaría* and *Oncomelania nosophora* also excreted cercaria e-cercariae	Development of sporocysts, two generations of radiae, cercaria requiring ∼3 months for the cycle. All stages are microscopic <0.7 mm)	Ameel (1934); Ameel et al. (1951); Basch (1959); Harley (1972); Lloyd et al. (2015)
Second intermediate host	Crayfish (*Orconectes* spp.)	∼ 2 months for metacercariae maturation	Ameel (1934); Stromberg et al. (1978); Harley (1972); Fischer et al. (2011)
Egg excreted	Eggs operculated, 78–97 × 46–60 μm in freshly excreted feces	Eggs excreted >30 days after ingestion of metacercariae by definitive hosts	Dubey et al., 1978a, Dubey et al., 1979a
Metacercaria	Glistening white∼0.5 mm in diameter, in heart of crayfish	Metacercariae can live for ∼1 year. The internal transcribed spacer (ITS2), 28S ribosomal RNA and other genes characterized (Fischer et al., 2011)	Ameel (1934); Stromberg et al. (1978); Fischer et al. (2011)
Migration in definitive hosts	After oral ingestion, fluke penetrates small intestine to reach peritoneal cavity, penetrate diaphragm, and enters lung through pleural surface. Worm matures in ∼4 weeks	Most parasite growth occurs after penetration of lungs	Stromberg and Dubey (1978)
Mature fluke	Adults ∼15 mm long, 7 mm wide, 6 mm thick and weigh ∼1 g. Lives probably for the life of host. Lays 1000–2000 eggs per day per fluke		Ameel (1934); Stromberg and Dubey (1978)

Paratenic hosts are optionally involved in the life cycles of certain species of *Paragonimus* prevalent in the Far East (Blair, 2022). Paratenic hosts are those where *Paragonimus* fails to develop to adulthood after the ingestion of metacercaria-infected crustaceans but remains viable as juvenile. Examples of paratenic hosts are deer, rodents, and pigs (Blair, 2022). The carnivorous host can become infected by ingesting tissues of paratenic hosts. An example of such a case is the recent report by Nakashima et al. (2021) of a 60-year-old man who developed clinical paragonmiasis after eating meat of a wild pig in Japan. There is no evidence for the paratenic hosts in the life cycle of *P. kellicotti* and the infected crayfish is the sole source of infection for mammals.

Until 1970, studies of *P. kellicotti* focused on life cycle stages and occurrence in various hosts. Little was known of clinical disease, pathogenesis, or diagnosis. I (J.P.D.) was appointed in 1973 at the Department of Veterinary Pathobiology Ohio State University. Columbus, Ohio (OSU) to teach parasitology to veterinary students. No teaching materials were then available in the Department of Pathobiology concerning fluke infections in cats or dogs. I had researched coccidian parasites, including *Toxoplasma,* but had never researched flukes. My opportunity to study *P. kellicotti* infection was facilitated by the availability of parasite-free cats and dogs raised under laboratory conditions; these were first obtained in 1960's in that department. Willing collaborators facilitated success, including Drs. Edward Hoover (pathologist), Paul Stromberg (zoologist-parasitologist, who was enrolled as veterinary student), Robert Pechman (radiologist), Surrender Sharma (clinician), and 2 technicians (M. Toussant, T. Miller). Conducive facilities to house cats and dogs in a research wing next to the veterinary hospital meant that medical attention was at hand. The Ohio Canine and Feline Research Funds supported the effort. A nearby woodland area stream provided easy access to infected crayfish. Thus, we could perform extensive studies of the life cycle, pathogenesis, clinical disease, diagnosis and develop treatment for *P. kellicotti* infection in experimentally infected cats and dogs not previously exposed to this parasite (Dubey et al., 1977a, Dubey et al., 1977b, Dubey et al., 1978a, Dubey et al., 1978b, Dubey et al., 1979a, Dubey et al., 1979b; Hoover and Dubey, 1978; Stromberg and Dubey, 1978; Stromberg et al., 1978). Previous life cycle studies had mostly been performed in animals supplied by animal contractors with no knowledge of prior exposure or health histories. *Paragonimus* infections were diagnosed in cats and dogs obtained from Animal Shelters or supplied by licensed suppliers (Alden et al., 1980; Stewart et al., 1981; Kern, 1991). I was also curious to understand whether stress induced by *Paragonimus* infections might induce chronicallyinfected cats (these cats excreted oocysts during acute primary infection) to re-excrete oocysts of *Toxoplasma gondii* without reinfection from outside (relapse) (Dubey, 1976). *Paragonimus* infection is not directly transmissible to cats housed in the adjacent cages, thus providing control for cage- to- cage transmission.

The recent reports of clinical paragonimiasis in Americans who had eaten raw crayfish rejuvenated my interest in *P. kellicotti* infection. Several unanswered questions remain regarding human infections. For example, do such infections provoke cutaneous rash and neural symptoms observed in some human patients? (reviewed in Coogle et al., 2022); no such lesions were described in animals experimentally or naturally infected with *P. kellicotti*. Further, is crayfish ingestion the sole route of human infection? Pigs and goats and other livestock have been mentioned as host for *P. kellicotti (*Coogle et al., 2022) but no such valid report of *P. kellicotti* infection in livestock species have surfaced in the last five decades. As stated earlier, paratenic hosts are unknown in the life cycle of *P. kellicotti*. Thus, livestock consumption appears very unlikely to pose any risk to people. Here, I have reviewed these old reports of *Paragonimus* infections in pigs and a goat in USA.

The objective of the present review is to summarize information on paragonimiasis in animals and humans in the USA, in particular pathogenesis in experimentally infected cats and dogs performed in 1970's. I have attempted to consult and summarize original papers on *Paragonimus* infections here to clarify misinterpretations. Although there are 66 records of submissions of *Paragonimus* infections listed in the United States National Parasite Collection (USNPC) (previously at the Animal Parasitic Diseases Laboratory, Beltsville, Maryland, now at the Department of Invertebrate Zoology, Smithsonian Institution, National Museum of Natural History), it is often difficult to match records with their publications. Therefore, I have listed the USNPC number when stated by the authors. Additionally, I now deposit immature and mature specimens of flukes, histologically stained sections of tissues with lesions, and a paraffin block of infected lung in the Smithsonian Museum and provide museum numbers for the benefit of future researchers.

## 3 Life cycle of *P. kellicotti*

**Fig. 1:**
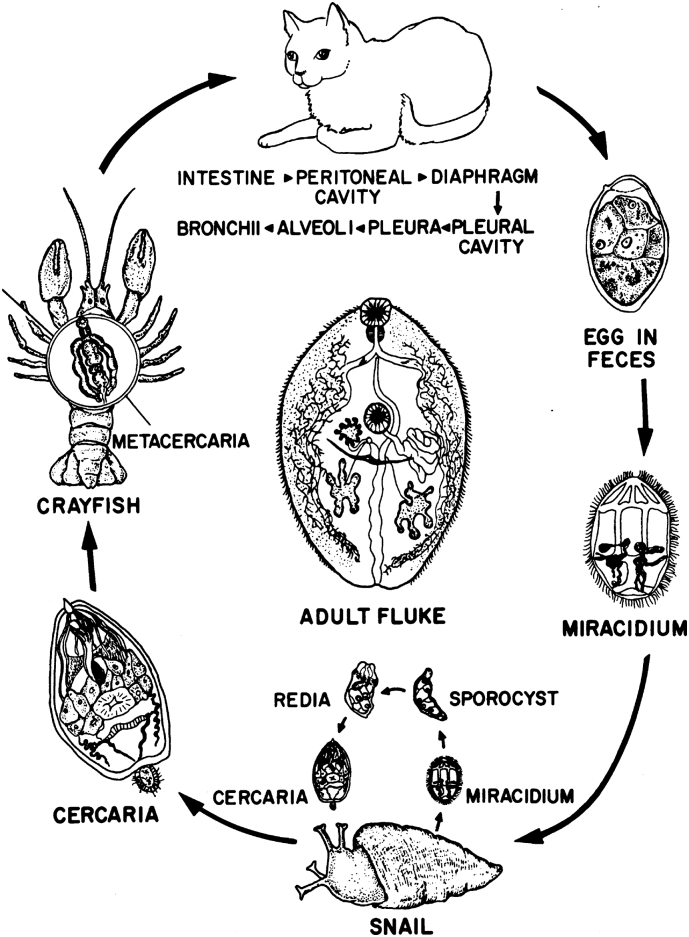
Life cycle of *Paragonimus kellicotti* in cats. (From Dubey et al., 1978a).

**Fig. 2:**
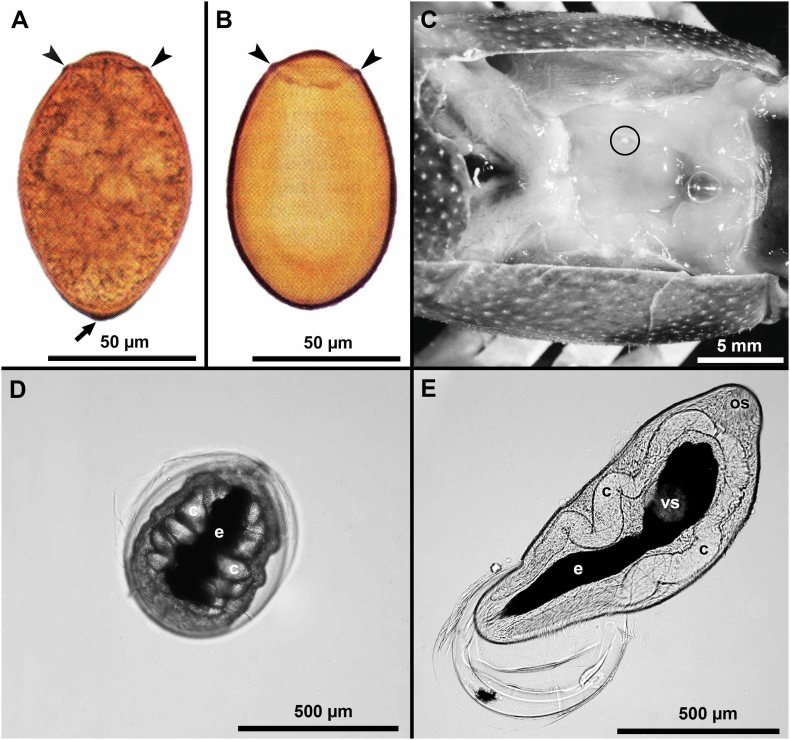
Environmental stages of *Paragonimus kellicotti.* (A, B) Eggs in feline feces, using sedimentation technique (A) and salt flotation technique (B). Unstained. Eggs are multicelled, operculated (arrowheads), and golden brown. There is a thickening (arrow) at the pole opposite the operculum. *Paragonimus* eggs become distorted in hypertonic salt solution, leaving only the eggshell. (C) Crayfish (*Orconectes* sp.), the second intermediate host of *P. kellicotti*, dissected to show the white gelatinous heart. Metacercaria (circled) is embedded in the heart of crayfish. (D) Metacercaria removed from the crayfish. Note dark excretory system (e) and the white ceca (c). (E) Young fluke squeezed out of metacercaria. Note oral (os), ventral sucker (vs, partly obscured), excretory vesicle (e), and 2 ceca (c). (Figs. A, B, C from Dubey et al., 1978a; Figs. D and E, courtesy of Dr. Peter Fisher, University of Washington, St. louis, Missouri, USA). (For interpretation of the references to colour in this figure legend, the reader is referred to the web version of this article.)

Information on life cycle stages, and hosts is summarized in Table 1, Table 2, Table 3 and Fig. 1, Fig. 2. *Paragonimus kellicotti* eggs are excreted in feces of definitive hosts in multi-celled stage. Depending on environmental conditions, it takes several weeks for the miracidium to develop and eggs to hatch, probably requiring flooding because definitive hosts (mink, *Mustela vison*) defecate on land. Cillia on the surface of miracidium propel it towards the freshwater snails. Ameel (1934) studied its development in freshwater snail, *Pomatiopsis lapidaria*. However, this snail was not found naturally infected in Missouri; instead, another snail, *Elimia potosiensis,* was found commonly infected with *P. kellicotti* as confirmed by PCR (Lloyd et al., 2015).

**Table 2:** Selected reports of *Paragonimus kellicotti* infection in wildlife in North America.

Year surveyed	Location	Prevalence	Comment	Reference
1929–1933	Michigan, Ohio, USA	Mink (*Mustela vison*), 94 of 588 (16%), Muskrats (*Ondantra zibithicus*),18 of 369 (4.8%), 0 of 109 opossums (*Didelphis virginiana*), 0 of 308 raccoons (*Procyon lotor)*		Ameel, 1931, Ameel, 1934
1940	Tennessee	Opossum (*Didelphis virginiana*), 1 of 3		Byrd (1941)
1932–1944	Minnesota, USA	Mink, 2 of 79 (2.5%)		Erickson (1946)
1956–1957	Georgia, USA	Opossum (*Didelphis marsupialis*) 39 of 96, raccoon 7 of 32 (21.8%), striped skunk (*Mephitis mephitis*) 6 of 55(10.9%), wild cat (*Felis rufus?*) 1 of 6 (16.6%)	Flukes twice as bigger (14.6 × 6.2 mm) in skunk than in other hosts	McKeever (1958)
1957	Georgia, USA	Bobcat (*Lynx rufus*)	1 adult 14.8 long × 6.6 mm wide	Jordan and Byrd (1958)
1960–1961	Ohio, USA	Mink, 22 of 62 (35.4%) from 7 counties	Metacercariae were found in 216 of 489 (44.7%) crayfish from Portage County	Gesinski et al. (1964)
1969	Minnesota, USA	Skunk (*Mephitis mephitis*), 1 of 1		Bemrick and Schlotthauer (1971)
Not stated	Kentucky, USA	Muskrat, 2 of 115 (1.7%), 0 of 15 field mice, 0 of 4 red fox, 0 of 4 raccoons, 0 of 3 dogs and 0 of 2 cats		Harley (1972)
1972	Michigan, USA	Red fox (*Vulpes vulpes*), 4 of 39 (10.2%)	Only 1 cyst per fox	Stuht and Youatt (1972)
1972–1974	Ontario, Canada	Mink, 16 of 105 (15.2%), striped skunk, 14 of 244 (5.7%), red fox, 10 of 446 (2.2%), coyote (*Canis latrans*), 1 0f 31 (3.2%), Raccoon, 0 of 323	Lesions described by Ramsden and Presidente (1975)	Ramsden and Presidente (1975)
1981–1982	Louisiana, USA	Opossum, 1 of 2		Shoop and Corkum (1982)
1985–1986	Kentucky, USA	Raccoons, 15 of 70 (21.4%)	Flukes deposited in Manter Lab^a^	Cole and Shoop (1987)
1989–1990	Arkansas, USA	0 of 30 raccoons		Richardson et al. (1992)
1972–1989	West Virginia, USA	Gray fox (*Urocyon cinereoargentineus*) 1 of 7 (14%)		Davidson et al. (1992)
2003–2012	Illinois, USA	Bobcat, 4 of 67 (6.0%)	USNPC no. 106450^b^	Hiestand et al. (2014)

a Manter Laboratory, University of Nebraska State Museum.

b United States National Parasite Collection.

**Table 3:** Prevalence of *Paragonimus kellicotti* metacercariae in crayfish in 2 studies from Ohio and Missouri, USA.

Region	Time period	No. of crayfish surveyed	No infected (%)	Average per crayfish (range)	Reference
Ohio^a^	July 1975–May 1977	796	467 (58.6)	3.0 (1−13)	Stromberg et al. (1978)
Missouri^b^	April–September 2010	144	93 (65)	2.8 (1–13)	Fischer et al. (2011)

a In municipal park 41.8% of 347 crayfish, and in woodlot 71.7% of449 crayfish were infected.

b Prevalences in crayfish from 3 rivers were: 69% of 16 from Big Piney, 36.8% of 19 crayfish from Black River, and 66% of 109 crayfish from Huzzah River.

*Paragonimus kellicotti* multiples in the snail as sporocyst, two generations of radiae, and finally cercariae; development in snail requires around three months (Ameel, 1934). Cercariae released from the snail penetrate the second intermediate host, crayfish belonging to at least two families (Table 1). A stylet on the *P. kellicotti* cercaria helps to penetrate tissues of crayfish and develop into metacercariae; maturation of metacercariae requires around two months (Ameel, 1934). Farm-bred crayfish are not considered a source of *P. kellicotti* infection.

**Fig. 3:**
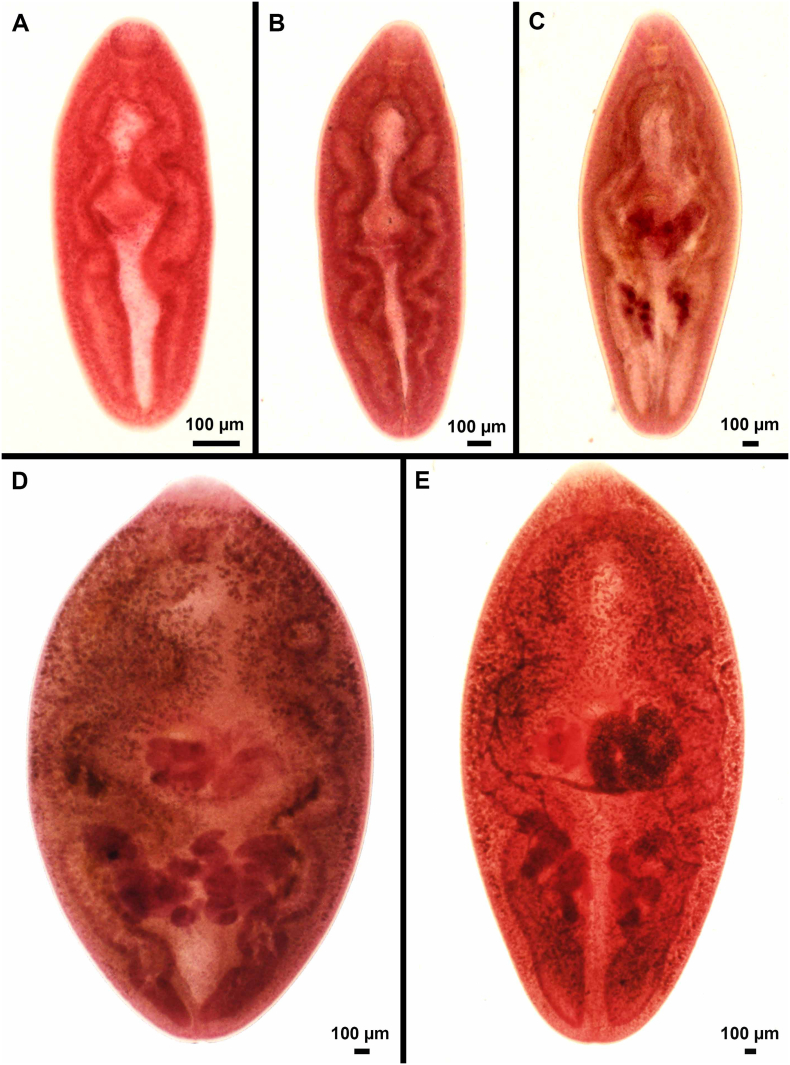
*Paragonimus kellicotti* stages recovered from experimentally infected cats. Flukes were fixed under moderate pressure and stained with Semichon's carmine as described (Stromberg and Dubey, 1978). (A) Day 7 p.i. (B) Day 14 p.i. (C) Day 21 p.i. (D) Day 29 p.i. (E) Day 34 p.i. Flukes at days 7 and 14 p.i.appear morphologically similar.

Population biology of *P. kellicotti* metacercariae has been studied in Ohio and Missouri (Table 3). It is remarkable that although there were some geographical/ habitat variations, in both studies around 60% of crayfish were infected with a similar parasite load, averaging 2–3 metacercariae per crayfish (Table 3). In two habitats (municipal park, woodlot) in Columbus Ohio, prevalence was 41.8% in a municipal park versus 71.7% in the wood lot; this was expected because woodlot will be visited more often by wild definitive hosts (such as mink) than the municipal park. Metacercariae occurred year-round (Stromberg et al., 1978). The metacercariae were identified morphologically and by bioassay in cats and dogs. Metacercariae recruitment in crayfish occurred during summer and autumn, metacercariae lived up to a year, but some died and were mineralized. Prevalence and intensity of infection increased with body weight of crayfish (Stromberg et al., 1978). Microscopic examination of infected crayfish revealed no inflammation.

**Fig. 4:**
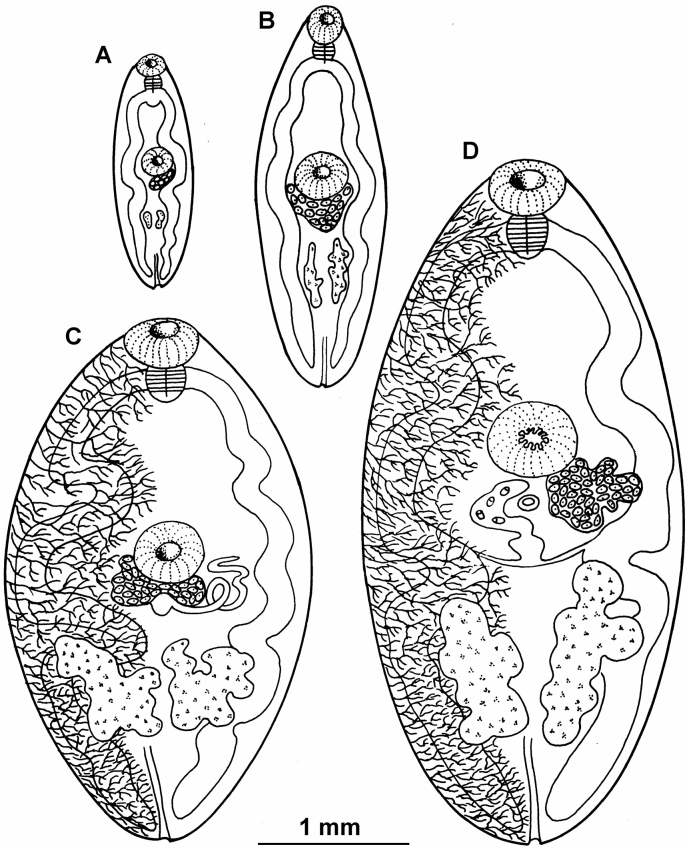
Line drawings of *Paragonimus kellicotti* from experimentally infected cats. The drawings were made from flukes shown in Fig. 3. Fluke at day 7 p.i. is like the fluke released from the metacercaria (not shown). (A) Fluke at day 14 p,i., female genital appeared just below ventral sucker (also called acetabulum) and testicular primordia were seen towards the excretory pore. (B) Fluke at day 21 p,i, main vitelline ducts have formed. (C) Fluke at day 29 p.i., the tubular uterus, and shell glands formed. (D) Fluke sat day 34 p.i., a few eggs had formed. (From Stromberg and Dubey, 1978).

The survey in Missouri was conducted for a period of six months in 2010 (30 years after the study in Ohio) but with similar prevalence rates (Table 3). Floating streams in three rivers were tested for metacercariae. The metacercariae were identified morphologically and molecularly; 65% of crayfish were infected (Fischer et al., 2011).

In a survey of intermediate hosts in Kentucky, USA, *P. kellicotti* cercariae were found in 3 of 18 *Pomatiopsis lapidaria* and 9 of 22 *P. cincinnatiensis* freshwater snails and metacercariae were detected in 24 (12%) of 200 *Orenectes juvenilis* and 1 of 28 *Cambarus bartoni* crayfish. As many as 32 metacercariae were found in an infected crayfish (Harley, 1972).

## 4 Experimental infections in parasite-free cats and dogs

### 4.1 Summary of experimental *P. kellicotti* infections in cats and dogs at OSU

#### 4.1.1 Cat

In total, 28 cats were inoculated orally with 14–50 metacercariae and observed for up to 263 days. The dosage of metacercarae used to infect cats was modest considering that up to 32 metacercariae can be present in a single crayfish as stated above (Harley, 1972). Clinically, eight cats were observed for >12 weeks. For blood and biochemical evaluation (total leukocyte counts, differential leukocyte counts, hemoglobin, packed cell volume, and total protein), cats were tested weekly for 12 weeks (Dubey et al., 1978a).

All cats were euthanized at different intervals. Complete necropsies were performed, and their internal organs were studied histologically. Lungs were fixed with airways perfusion with 10% formalin. For migratory pathway and pathogenesis, cats were euthanized 1, 3, 5, 7, 10, 14, 21, 23, 29, 34, 39, and 55–263 days later (Table 4). Tissues were embedded in paraffin, cut at 3–5 μm thick, and examined microscopically after staining with hematoxylin and eosin (HE) stain. The following observations were made.

**Table 4:** Migration, fluke development, and lesions in experimental *Paragonimus kellcotti* infection in cats (data from Hoover and Dubey, 1978; Stromberg and Dubey, 1978; Dubey et al., 1978a)).

P.i. day	No. of cats	Flukes found	Fluke size and development	Lesions
1,3	2	Peritonium	<1 mm long	None
5	1	Peritonium, pleura	<1 mm long	10 ml of fluid in peritoneum, eosinophilic peritonitis
7	3		<1 mm long (Fig. 3A). Cercarial stylet present	
10	2	Peritonium, pleura	<1.5 mm long. Cercarial stylet absent. Female genitalia developing	Eosinophelic pleuritis. Fibrinous plaques associated with penetration of pleura (Fig. 5A)
14	5	Peritonium, pleura. Flukes <1.5 mm long.	<1.5 mm long. Cercarial stylet absent. Testis developing. (Figs. 3B, 4A)	Focal hemorrhages, eosinophilic pleuritis, fibrinous plaques
21	1	Pleura and lung	2.0–2.5 mm long. Vitelline ducts formed (Figs. 3C, 4B)	Bright red, 6–15 mm diameter hemorrhages
23	2	Lungs	∼3 mm long. Uterus and shell gland formed.	Bright red, 6–15 mm diameter hemorrhages (Fig. 8A)
29	1	Lungs	∼3 mm long. (Fig. 3D). Vitellaria proliferation	Red brown cystic lesions, 10–15 mm in diameter (Fig. 8B)
34	1	Lungs	∼3 mm long. (Figs. 3E, 4D). Few eggs visible in uterus	Red brown cystic lesions, 10–15 mm in diameter (Fig. 6A)
39	1	Lungs	Mature flukes	Reddish gray cystic lesions in lungs. Cysts communication with bronchioles. (Fig. 6B) Pneumothorax due to rupture of a cyst.
55–263	4	Lungs	Mature flukes. Cystic nodules	Cystic nodules (Fig. 8C,D)

##### 4.1.1.1 Migratory pathway and life cycle

After ingestion, excysted flukes were found in peritoneal washing by 24 h post-inoculation (p.i.). Flukes reached pleural cavity by penetrating diaphragm and were found in pleural cavity as early as day 4 p.i. The migration was not synchronous because some flukes were present in peritoneal cavity on day 14 p.i. and some flukes were free in pleural cavity on day 23 p.i. (Stromberg and Dubey, 1978). As many as 87% reached the lungs; in 1 cat, 13 adults were recovered on day 263 after feeding 15 metacercariae. More flukes were found in caudal lobes of lungs, adjacent to diaphragm than in other lobes. Most growth of the flukes occurred in the lungs. Flukes reaching the lungs were usually <3 mm long; adult flukes were as large as 15 mm. Mature adult flukes weighed ∼1 g.

Eggs were first detected in feces of a cat day 34 p.i. the cat was fed 14 metacercariae (Dubey et al., 1978a). Eggs per gram of feces (epg) was monitored daily in six cats, 70–85 days after consuming metacercariae. All feces voided in 24-h were weighed and emulsified in two volumes of water. Because the egg excretion is not continuous, epg was estimated in total daily feces (Stromberg and Dubey, 1978). It was estimated that each fluke excreted 1000–2000 eggs daily. In one cat, egg count was 1350 on day 80, 920 on day 90, 1100 on day 100; thereafter egg counts were stable until the cat was euthanized day 153 (Dubey et al., 1978a). The sedimentation technique was more efficient for the diagnosis of eggs in feces; eggs were detected eight days earlier by the sedimentation technique than by the McMaster technique (Dubey et al., 1978a). For this, feces of cats voided days 36–70 p.i. were tested by both techniques; no eggs were detected by the McMaster technique using feces days 36–43 p.i. when the egg count by sedimentation technique was low (40–180 epg). Both techniques detected eggs in feces of cats days 44–70 p.i. (Dubey et al., 1978a).

##### 4.1.1.2 Pathogenesis

**Fig. 5:**
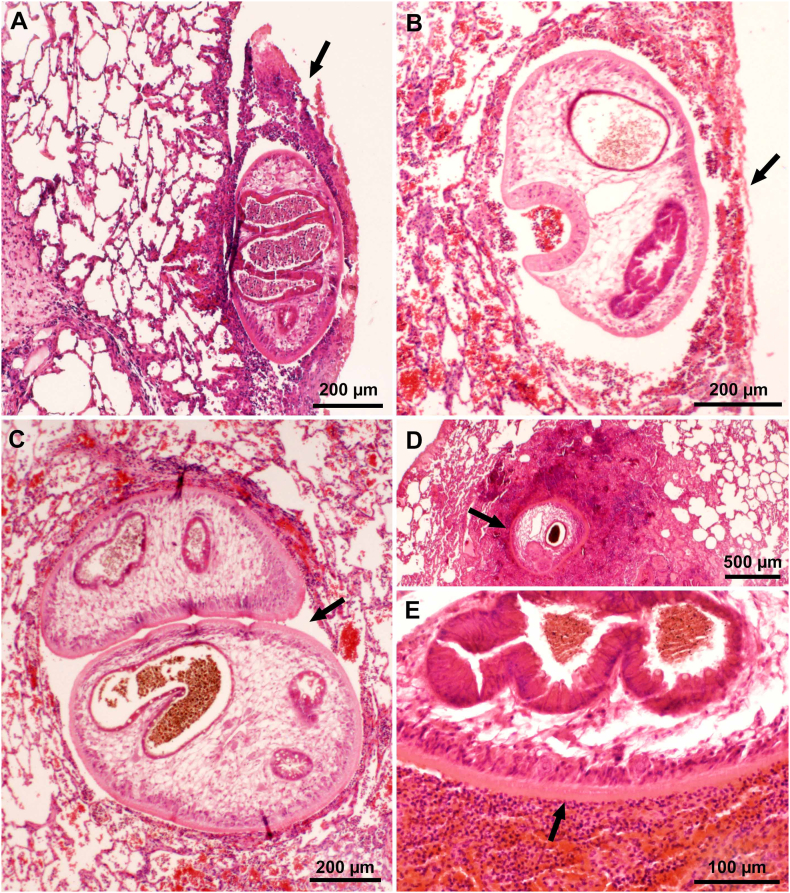
Pathogenesis of pulmonary paragonimiasis in experimentally infected cats. Histological sections of lungs. HE-stain. (A) Penetration of pleura by immature fluke. Note inflammatory exudate (arrow) with fibrin, eosinophils, extravasated erythrocytes covering the fluke. Day 10 p.i. (B) Recently penetrated fluke in subpleural cavity of pulmonary parenchyma. The pleural breakage (arrow) is healing. The fluke is feasting on host tissue. Day 14 p.i. (C) Paired immature flukes within lung parenchymal cavity (arrow). (D, E) Immature fluke with intense eosinophilic inflammation and necrotic tissue (arrows) surround the fluke. Day 21 p.i. (From Hoover and Dubey, 1978).

**Fig. 6:**
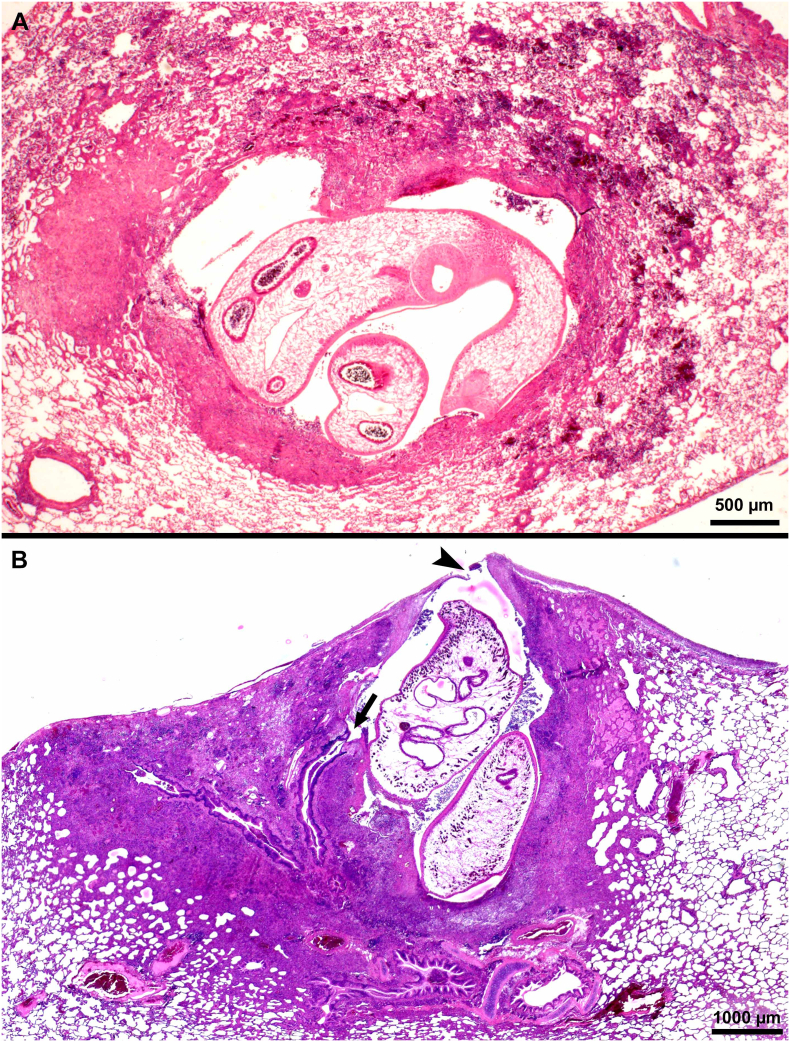
Cystic lesions in lungs of cats fed *Paragonimus kellicotti* metacercariae. Histological sections of lungs. HE-stain. (A) Early stage of cyst formation. Two immature flukes are surrounded by hemorrhagic exudate containing eosinophils and other inflammatory contents. Day 29 after feeding 25 metacercariae. (B) A cyst with a very thin or broken pleural surface (arrowhead) and a communication with bronchus (arrow). Two mature flukes are surrounded by fibrous tissue, exudate, and granulomatous tissue. Eggs are present in surrounding tissue but not visible at this magnification. Day 39 after feeding 34 metcercariae.

**Fig. 7:**
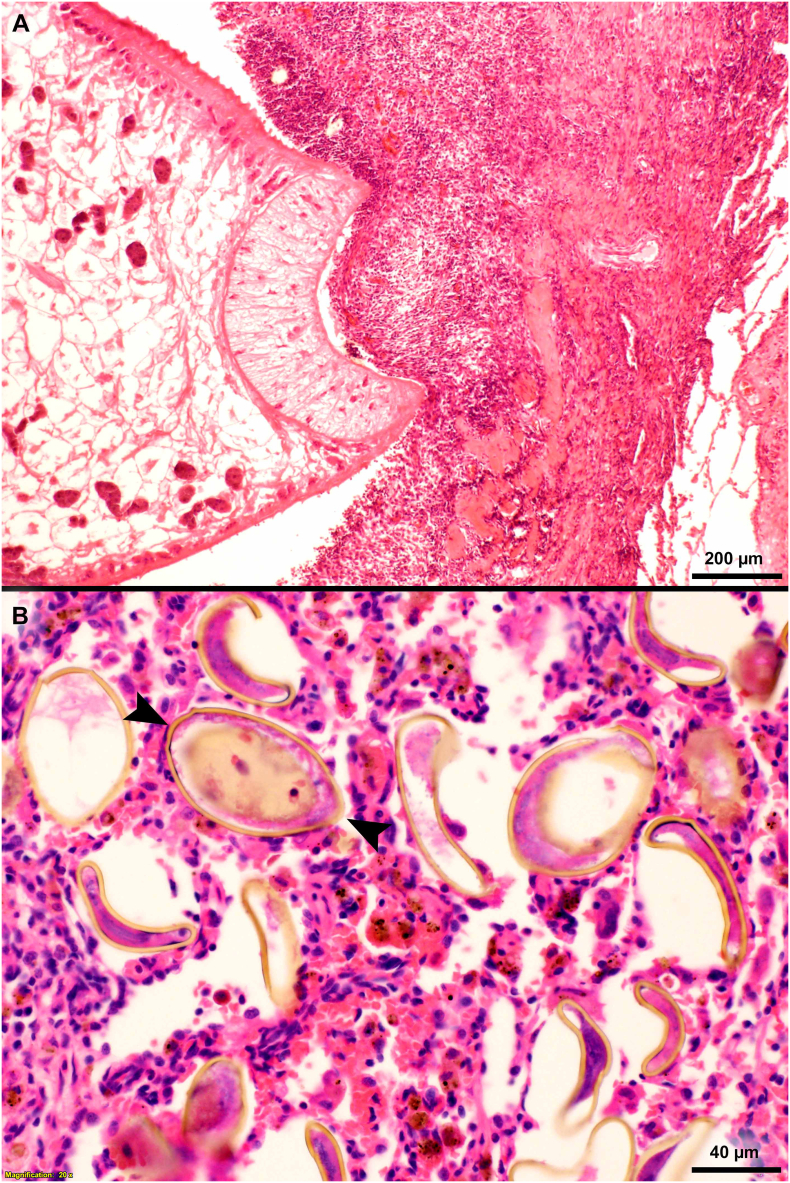
Microscopic appearance of *Paragonimus kellicotti* in histological sections of lungs. HE-stain, day 70 after feeding 25 metacercariae. (A) Cat. Anterior end of a fluke attached to granulomatous tissue. The cuticular spines of the fluke probably irritate the host tissue provoking granulomatous response. (B) Dog. Several golden-brown eggs are trapped in alveolar tissue. One of the eggs is cut longitudinally (opposing arrowheads) with operculum at one end and a slight thickening at the narrow end. (For interpretation of the references to colour in this figure legend, the reader is referred to the web version of this article.)

**Fig. 8:**
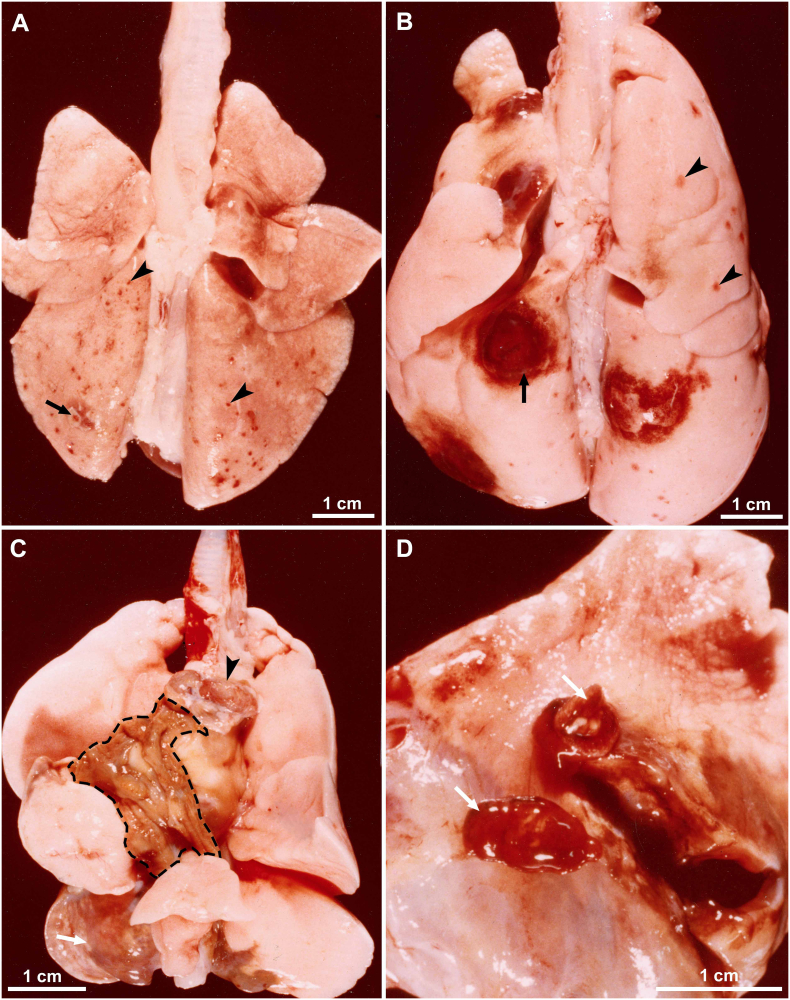
Gross appearance of lesions in lungs of cats fed *Paragonimus kellicotti* metacercariae. Unstained. (A) Early hemorrhagic lesions in a cat day 23 after feeding 50 metacercariae. More lesions are present in caudal lobes (near penetration site in diaphragm) than in other lobes. The number of hemorrhagic spots (arrowhead) exceed the number of metacercaria fed indicating repeated penetration of flukes searching for a site to settle. Note one pleural plaque (arrow), probable the site where fluke has successfully penetrated. (B) Dorsal view of early hemorrhagic appearance of cysts (arrow) in a cat day 29 after feeding 50 metacercariae. Arrowheads point to early penetration sites. (C) Ventral view of lungs of a cat 56 days after feeding 26 metacercariae. Bronchial lymph node (arrowhead) is enlarged, the mediastinum is colored brownish (shaded area) due to eggs, and cysts are also greyish (arrow). (D) Incised cyst revealing two flukes (arrows) in a cat day 263 after feeding 15 metacercaria (From Dubey et al., 1978a).

**Fig. 9:**
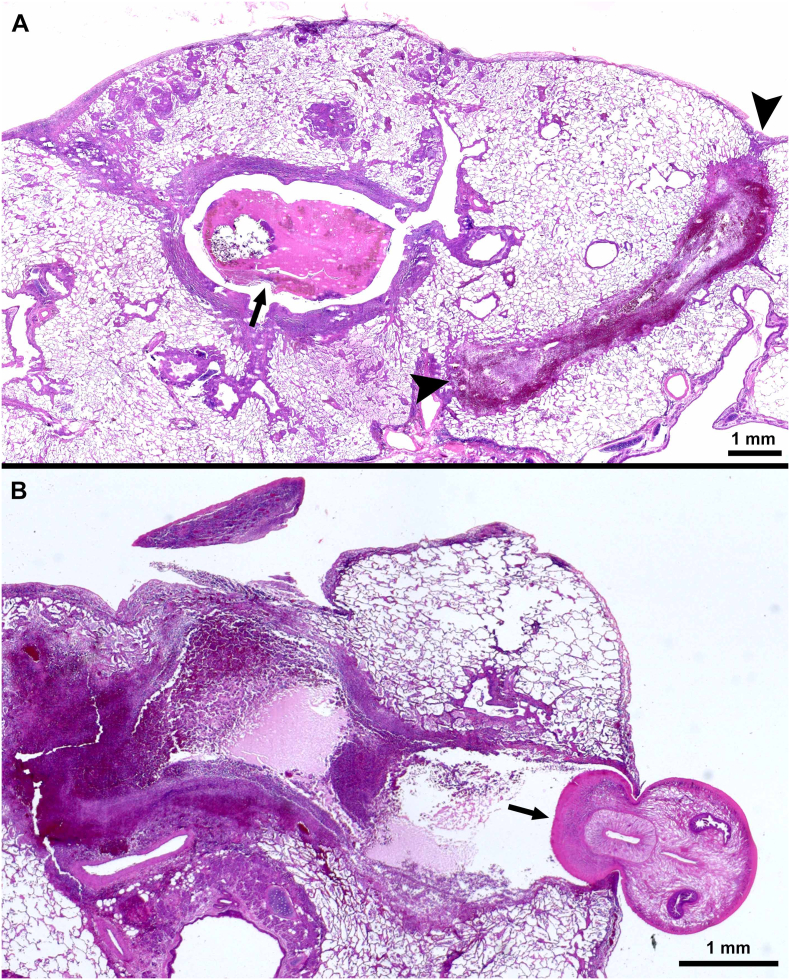
Lungs of a *P. kellicotti* infected cat, 21 days after treating with albendazole (50 mg/kg body weight). Treatment of cat was started on day 101 after feeding 25 metacercariae and therapy was continued for 21 days. HE-stain. (A) Note a dead fluke (arrow) and a hemorrhagic tract (opposing arrowheads) indicating probably the tract left by a fluke that has existed from the lung. (B) A fluke (arrow) exiting from pleura.

Observations are summarized in Table 4. The lesions were primarily found in lungs and the pleural cavity. (Fig. 5, Fig. 6, Fig. 7, Fig. 8, Fig. 9).

##### 4.1.1.3 Peripheral eosinophilia

Two peaks of eosinophilia were observed (Dubey et al., 1978a). The first peak of eosinophilia occurred around three weeks p.i. and coincided with penetration of flukes in the lungs. The second occurred around eight weeks p.i., probably related to entrapment of eggs in the lungs and in the mediastinum.

##### 4.1.1.4 Radiographic changes

Earliest lesions were detected in caudal lobes of lungs weeks 2–3 p.i. (Dubey et al., 1978a). Early lesions were nodular with 2–4 cm air cavities with chambers divided by septa. By day 65 p.i., well defined cysts were formed. Pneumothorax was seen in four cats, in two of them recurrent. In one cat an entire lobe was consolidated (Dubey et al., 1978a).

##### 4.1.1.5 Clinical signs and outcome of infection

In general cats developed only mild disease. Coughing was the predominant sign. One cat had paroxysms of coughing when disturbed but otherwise appeared normal. One cat was euthanized because of dyspnea; pneumothorax was conformed at necropsy examination. One pregnant cat successfully delivered and nursed four kittens; *Paragonimus* infection was not detected in kittens at necropsy (Dubey et al., 1978a).

##### 4.1.1.6 Chemotherapy

Until 1976, bithionol was the only drug tried to treat natural paragonimasis in cats (Table 6). Bithionol, a phenol derivative, is relatively toxic. Benzimidazole derivative drugs are broad spectrum anthelminthic drugs which were commonly used in 1970's and 1980's. Albendazole was found effective in treating experimentally infected cats (Dubey et al., 1978b). Five cats fed 25 *P. kellicotti* metacercariae were medicated with albendazole (2 cats-20 mg/kg/daily, two cats 100 mg/kg daily, one cat 50 mg/kg daily) as oral suspension for 14–21 days. Egg excretion was reduced or stopped within one week of dosing drug; at necropsy performed 14–20 days after medication revealed shrunken or dead flukes and resoulation of lesions.(Fig. 9).

### 4.2 Dog

Thirteen dogs were euthanized between days 7 and 70 after feeding 25 or 50 metacercariae and same parameters investigated for cats were investigated for dogs (Dubey et al., 1979a, Dubey et al., 1979b). The life cycle and lesions observed in dogs were essentially similar to those in cats. Salient features of infections in dogs were:

(i). Free flukes were found in pleural cavity up to day 43 p.i.

(ii). Eggs were detected as early as day 30 p.i. in a dog.

(iii). Overall clinical disease was more severe in dogs versus cats. Two of five dogs dosed with 35 metacercariae died of paragonimiasis on the 32nd and 35th day p.i. One dog died of dyspnea suddenly on day 32 p.i. The second dog also died despite prompt clinical intervention and died despite being put on a respirator. Granulomatous pneumonia associated with *Paragonimus* eggs predominated in a dog, 70 days p.i. (Fig. 9B).

(iv). Lesions were found on liver day 7 p.i. and immature flukes were found in lesions by histological examination of stained sections. Focal hepatitis persisted until day 35 p.i. Fibrinous deposits were detected on the serosal surfaces of kidneys, spleen, and other visceral organs but flukes were not Found in lesions.

(v). Eosinophilic myositis was detected in diaphragms days 7–24 p.i.

(vi). Fenbendazole (50 or 100 mg /kg body weight, orally) was found effective in treating infected dogs without side effects. Egg excretion stopped day 3 (100 mg/kg body weight) or day 3–8 (50 mg/kg) dosing with fenbendazole; therapy was started 6–7 weeks after dosing with metacercariae (Dubey et al., 1979a).

### 4.3 Infections in cats and dogs performed at Cornell University

Seven cats and seven dogs were inoculated with 12–22 metacercariae, and five cats and five dogs were treated with praziquantel (23 mg/kg of body weight, 3 times daily for 3 days), 7 or 14 weeks p.i. (Bowman et al., 1991). None of the dogs or cats developed visible clinical signs. Only five cats developed patent infection and eggs were detected in feces by seven weeks after infection. In dogs, pulmonic lesions were detectable days 21  p.i., and all lesions resolved by two weeks post treatment. Lesions were still detectable in cats by four weeks p.i. In treated dogs necropsied four weeks p.i., no flukes were seen but eggs were detectable in lungs and appeared morphologically normal (Bowman et al., 1991).

## 5 Natural infections in animals

### 5.1 Natural hosts of *P. kellicotti*

Although several carnivores are natural hosts in USA and Canada, mink (*Mustela vison*) is considered as one of the most important reservoirs of *P. kellicotti* infection (Table 1). Because there is uncertainty concerning the *Paragonimus* specimens from livestock (goats, pigs), I have reviewed the original publications.

There is an old record of *Paragonimus* infection in a goat (Hall, 1925). The goat in question, a year-old female, was slaughtered December 1924 at the National Stock Yards, Illinois and probably came from Mississippi (Hall, 1925). Thirty-six flukes recovered from lung were partly macerated and no spines were detectable on the cuticle; thus, it is unlikely that the fluke was *P. kellicotti*. This information was presented at a meeting; I could find no evidence that these flukes were subjected to further study.

Whether pigs are a host for *P. kellicotti* is also uncertain. Stiles and Hassall (1900) first recorded it. An unknown number of flukes collected from hogs (number not stated) were sent to Stiles by a meat inspector in charge of slaughterhouse in Cincinnati, Ohio. Stiles and Hassall considered them as the same fluke from the dog and cat in USA; *P. kellicotti* had not yet been named. They reviewed the worldwide literature on paragonimiasis known until 1900. Nothing more is known of the flukes recorded in that 1900 report. Ameel (1934) mentioned using *P. kellicotti* eggs from lungs of a pig for life cycle studies but did not provide details concerning infected pig. Also, there are anecdotal reports of *P. kellicotti* infection from Georgia, USA summarized by Jordan and Byrd (1958). These flukes were found in sections of lungs of two pigs slaughtered at Atlanta, Georgia; the source of pigs is undetermined. Cooperrider (1952) listed *P. kellicotti* among the parasites found in Georgia, but no details were provided. Stewart and Jones (1959) found small immature flukes in lungs of a pig from Fitzgerald, Georgia that were considered *Paragonimus rudis* (=*Paragonimus kellicotti*, USNPC no. 49428).

Pulmonic lesions associated with *P. kellicotti* infections were reported in bobcats and other carnivores (Table 2). Lungs of two of five bobcats from Arkansas contained approximately 1 cm diameter nodules that contained *P. kellicotti* (Snyder et al., 1991). Ramsden and Presidente (1975) and Presidente and Ramsden, 1975 provided a detailed description of lesions associated with *P. kellicotti* in several species of carnivores from Canada (Table 2). The nodular lesions were found in all lobes of mink lungs. Lesions were essentially similar in mink, skunk, and red fox. The nodules in coyotes had thick walls. Although 26 of 323 raccoon lungs had pleuritis including adhesions, flukes were not found.

*Paragonimus. kellicotti* eggs have been detected rarely in feces of cats and dogs in the USA examined routinely for parasitism (Conboy, 2009;). Data from general population surveys are summarized in Table 5. Most of the animals were stray. The number of animals found infected was an underestimate because *P. kellicotti* eggs have a higher specific gravity than solutions often used for flotations. Sedimentation technique is more sensitive, but it is time consuming and rarely used for routine surveys (Dubey et al., 1978a).

**Table 5:** Prevalence of *Paragonimus kellicotti* eggs in feces of dogs and cats in USA.

Region	Survey period	Method	No. tested	No. infected	Notes	Reference
		**DOGS**				
Michigan		Zinc sulfate or NaCL flotation	123	1	Stray-Humane shelter	Worley (1964)
New Jersey	1959–1964	Zinc sulfate flotation	Not stated	1		Burrows and Lillis (1965)
New Jersey	Not stated	Zinc sulfate flotation	2737	0	Stray(?)	Lillis (1967)
Ohio	1976	Sucrose-flotation	500	0	From Humane shelter	Streitel and Dubey (1976)
Pennsylvania	1984–1991	Zinc sulfate flotation	8077	1	Veterinary Clinic	Nolan and Smith (1995)
Oklahoma	1981–1990	Fecal flotation	21, 583	Present, number not stated	Veterinary Clinic	Jordan et al. (1993)
Nationwide	1993–1994	Sucrose flotation	11,391	2	1 of 6458 feces, national, and 1 of 1941 Southwest	Blagburn et al. (1996)

		**CATS**				
New Jersey	1959–1964	Zinc sulfate flotation	Not stated	3		Burrows and Lillis (1965)
New Jersey	Not stated	Zinc sulfate flotation	1480	0	Stray(?)	Lillis (1967)
Ohio	1976	Sucrose-flotation	1000	0	From Humane shelter	Dubey et al. (1977a)
Pennsylvania	1984–1991	Zinc sulfate flotation	2000	0	Veterinary Clinic	Nolan and Smith (1995)

#### 5.1.1 Clinical *P. kellicotti* infections in animals

Clinical paragonimiasis associated with *P. kellicotti* infection has been reported in domestic cats and dogs in USA and Canada (Table 6). Salient features of these reports are indicated in bold. Most cases from Canada were from Ontario province and there were no reports of *P. kellicotti* infections from western parts of USA and Canada.

**Table 6:** Clinical paragonimiasis in domestic cats and dogs in USA and Canada.

Region	No	Observations	Reference
		**CATS**	
North Carolina	1	Two *P. kellicotti* in a cat, 1 free in pleural cavity and 1 embedded in lung; no other information available.	Hardcastle (1941)
Ontario, Canada	2		Nielsen (1955)
Iowa, USA	1	Six-year-old male, Story County, urinary calculi, persistent cough for t 6 months. Died suddenly, 3 cysts, each with 1 fluke in lungs.	Greve et al. (1963-1964)
Indiana, USA	1	Six-year-old male, from Lafayette, wheezing for 5 months, feces negative for *Paragonimus* eggs. Radiography revealed a 3-cm diameter dense area in diaphragmatic lobe. Exploratory **thoracotomy**. **Lobectomy of diaphragmatic revealed a 3 cm nodule that contained 3 adult flukes**. The cat recovered.	Bisgard and Lewis (1964)

Arkansas, USA	1	Mature male, **wheezing and coughing, sometimes with hemoptysis for 10 months**. *Paragonimus* eggs in feces, treated with drocarbil for tapeworms, **died of asphyxia,** pleural adhesions, several cysts with 1–3 adult flukes, bronchopneumonia at necropsy.	Herman and Holland (1966)
Ontario, Canada	5	Retrospective study of 4 of 5 cases since 1967, *Paragonimus* eggs in feces of all 5 cats. **Radiographically, soft tissue density was detected in diaphragmatic lobe in all 4 cases**. Case 1, 8-month-old male without symptoms. Case 2, 8.5 months-old female, coughing for 3 days. Died with Feline Infectious Peritonitis complications. Case 3, 5-year-old male, was coughing for >1 month. Case 4, 6-year-old female, bouts of coughing for 5 weeks, died of myelogenous leukemia. Case 5, 6.5-year-old female was coughing for 1 month. Treatment was performed in 1 cat (cat not specified). **The cat had single lesion in diaphragmatic lobe that was lobectomized**.	Rendano Jr. (1974)
Alabama, USA	1	Adult male, anorexia and vomiting, *P. kellicotti* eggs in feces. Treatment with niclosomide (1 g, Yomesan), twice the dose for tapeworm treatment, stopped shedding of *P. kellicotti* eggs.	Nance and Bailey (1975)
Ohio, USA	3	Retrospective review of cases of paragonimiasis at the Ohio State Veterinary Teaching Hospital (OSUVTH) between 1966 and 1974) for radiographic changes. Case 1, 3-year-old female from Reynoldsburg, coughing for 3 months, diagnosis confirmed by necropsy examination. Case 2, 5-year-old male from Columbus, dyspnea, no follow up. Case 3, 2-year-old male from Columbus, coughing for 1 year.	Pechman (1976)
West Virginia	1	*P. kellicotti* eggs in feces of **1 of 135 cats** obtained for experimentation **from a shelter** (Dog Pound) in Monongalia County. Cat mature male, sneezing periodically, handling induced respiratory distress. Radiography revealed non-cavitated soft tissue densities in lungs. Diagnosis confirmed at necropsythe diagnosis.	Alden et al. (1980)
Missouri, USA	1	Incidental finding in an adult **female cat used in laboratory for cytauxzoonosis research**. **Several dozen***P. kellicotti* flukes found in bronchioles at necropsy.	Stewart et al. (1981)
Indiana, USA	2	**Paragonimiasis in 2 cats successfully treated with albendazole (25 mg/kg, twice daily for 10 days**). Case 1, 3-year-old female, coughing, wheezing, diagnosed by fecal examination and radiographic findings. Case 2, 9-months-old female, hospitalized for persistent cough for 6 weeks, blood eosinophilia, *Paragonimus* eggs in feces, radiographic density in lung. After treatment, eosinophilia and eggs disappeared, and pulmonic lesions reduced.	Johnson et al. (1981)
Louisiana, USA	10	**Albendazole (50 mg/kg body weight, twice daily for 11–24 days) therapy was successful in 8 of 10 cats with paragonimiasis.** Cats, 2–5 years-old, cough for 1–9 months, paragonimiasis confirmed by fecal examination and radiography. **Treatment partially successful in a 2-year-old female medicated with albendazole for 23 days, euthanized because of pneumothorax, 16 flukes recovered from nodular lung lesions at necropsy. The other cat with failed therapy, a 2.5-years-old male, treated with albendazole for 24 days, eggs still present its feces, cat not necropsied.** After a few days of therapy, cats disliked the drug and were hyper salivating.	Hoskins et al. (1981)
Oklahoma, USA	1	2-year-old-neutered male **developed clinical signs 1 week after surgical removal of testis (orchiectomy**). Cat had dyspnea and polydipsia/polyurea. *P. kellicotti* diagnosed by radiography and fecal testing. **Treated successfully with fenbendazole (50 mg/kg body weight) for 14 days. Cat had not travelled outside of Oklahoma.**	Rochat et al. (1990a)
Kentucky, USA	1	8-year-old barn cat died after episodes of coughing and respiratory distress. Necropsy examination revealed nodules, primarily in diaphragmatic lobe. Adult *P. kellicotti* and eggs were detected histologically.	Swerczek and Lyons (2000)
Ontario, Canada	1	**This is the most extensive radiographic evaluation of fenbendazole therapy of paragonimiasis in a 16-month-old female cat.** Cat coughing 20–30 times daily, 7 nodules in lungs and *P. kellicotti* eggs in feces, fenbendazole (28 mg/kg, orally, twice daily for 21 days) given and followed for a year, clinical improvement day 9 post treatment (pt), cough returned on day 49 pt. Thoracic radiographs revealed that cavitated lesions seen initially were smaller, but a solid nodule was not cavitated, and *P. kellicotti* eggs were still present in feces,treated second time with same dose of fenbendazole as before. On day 94 pt., *P. kellicotti* eggs not found but nodules still present in lungs. Cat was evaluated clinically on days 135 and 221 pt. and condition was stable. A final examination on day 316 pt. revealed no *P. kellicotti* eggs in feces and the pulmonic lesions were smaller. **The authors discuss the possibility concerning differences among strains of *P. kellicotti* in different regions of Canada and the USA.**	Peregrine et al. (2014)
		**DOGS**	
Ontario, Canada	2	A 2-year-old coon hound from Waterloo, several attacks of cough since it was a pup, dyspneic, died of suffocation, 16 flukes in cysts in both lungs, and eggs in feces at necropsy. The second dog, littermate of the dog that died, had mild symptoms and eggs in feces.	Nielsen (1955)
Arkansas, USA	1	**Three months-old- pup** died of dyspnea of 3 days duration, no eggs in feces, radiography revealed a cyst in lung, died a few hours after radiography. Necropsy revealed 2 (5–7 mm × 3–5 mm) trematodes in a pulmonic cyst. **The authors thought that the parasite was *Paragonimus westermani* based on morphology of eggs***.*	Short and Hendrickson (1960)
Ontario, Canada	1	Five- month-old Golden Labrador female from London, Ontario was **ovariohysterectomized in a clinic** and sent home after 4 days. The next morning the dog was dyspneic, running around frantically, and died. **Necropsy examination revealed pneumothorax, collapsed lungs, and a ruptured bronchus**. The lungs had 3 nodules and 9 flukes.	Comfort and Axelson (1962)
Iowa, USA	4	Dog 1, 7-year-old male Labrador retriever and a US Army K-9 Corps, examined in 1948 because of convulsions, fecal examination revealed eggs of *Ancylostoma caninum* and *Paragonimus,***served in Orient and whether it acquired infection overseas is uncertain**. Dog 2, 7-year-old male coon, from Polk County,fecal examination revealed *Capillaria* sp. *A. caninum*, and *P. kellicotti* eggs. Large (1–3 cm diameter) nodules were found in both lungs and contained adult flukes. **In 1 lesion the fluke was free in the pulmonary parenchyma with hemorrhage and inflammation.** Dog 3 with no other information, examined at the clinic. **A discrete abscess containing 2 flukes was surgically removed from 1 lobe of lung**. Dog 4, 7-year-old tan hound from Dallas County, admitted to clinic for treatment of dog bite wound, *A. caninum* and *P. kellicotti* eggs in feces, treated for hookworms and sent home.	Greve et al. (1963-1964)
Indiana, USA	1	*Paragonimus* eggs found during fecal examination of a 2-year-old-mongrel female asymptomatic dog used for teaching. Radiographic examination revealed increased density in lung lobes. **Thoracotomy of the diaphragmatic lobe revealed a 6 cm diameter nodule with granulomatous reaction**. Dog recovered.	Bisgard and Lewis (1964)
Pennsylvania, USA	1	This case is unusual because it was an **incidental finding of *P. kellicotti* in a Beagle born and raised in a kennel for drug trials**. The dog was necropsied at the end of a trial. Necropsy examination revealed a cystic nodule with eggs.	Wilson and Lord (1965)
Iowa, USA	2	Two cases of paragonimiasis treated successfully **with bithionol** (20–40 mg /kg, twice daily for—days). Case 1, **3-month-old** male Boston terrier with *Paragonimus* eggs in feces, mild cough, 20 mg /kg of bithionol not effective, therefore dose increased to 40 mg. **Increased in eosinophilia after treatment interpreted to indicate reaction to dead flukes.** Case 2,9-year-old male Coonhound, treated with 45 mg/kg body weight, eggs not detected 5 days after treatment.	Greve (1969)
Ontario, Canada	1	Four-year-old female Collie from Guelph, intermittent cough for 6 months, pneumothorax, and atelectasis of right apical lobe by radiography. Examination of bronchiolar lavage revealed *Paragonimus* eggs. No treatment was needed.	Gillick (1972)
Mississippi, USA	1	*Paragonimus kellcotti* infection diagnosed in cat (no details provided) by detection of eggs in feces, radiographs, and by necropsy examination (no details).	Majune and Moore (1975)
Illinois, USA	1	Two-year-old female Husky, *P. kellicotti* infection diagnosed based on a routine fecal examination. Radiography revealed mild diffuse interstitial lung density. No respiratory signs were reported. Medication with Bithionol (100 mg/kg mixed in food every other day for 30 days) stopped shedding of *P. kellicotti* eggs.	Macy and Todd Jr. (1975)
Ohio, USA	6	Retrospective radiographic review of cases of paragonimiasis at OSUVTH, 1966–1974) for radiographic changes. Case 1, 3-year-old Cairn Terr female from Cleveland, cough, acute dyspnea. Case 2, 15-years-old Afghan Hound female from Columbus. Mammary gland tumor. Diagnosis confirmed at necropsy. Case 3, 4-years-old Coonhound male from Newark, cough for 4 months. **Successfully treated with** b**ithionol.** Case 4, 4-months old, mixed breed female from Columbus. Cough and acute dyspnea. **Treated with** b**ithionol and right lobectomy.** Case 5, 8-months-old German Shephard male from Columbus, acute dyspnea. **Treated with** b**ithionol and left lobectomy.** Case 6, 7-months old Boxer male from Bloomville, acute dyspnea. **Medicated with bithionol but euthanized and necropsy examination.**	Pechman (1976)
Georgia, USA	1	Two-years-old mongrel male from Atlanta from a shelter had been used for filarial research. The dog was euthanized, examined at necropsy as part of the filarial research. The dog was emaciated, dyspneic, and had intermittent cough. **All lobes of the lungs had nodules that contained a total of 59 flukes, up to 4 flukes per nodule. The flukes were considered *P. kellicotti*, primarily based on the host species. This is the most heavily infection diagnosed in a dog.** **This dog had extrapulmonary lesions with *Paragonimus* eggs**. *Paragonimus* eggs were found in granulomatous lesions in scrotum, spermatic cord, liver, and mediastinal lymph nodes. **This is only case with extra pulmonic lesions in dogs. The identity of the *Paragonimus* species is in question**.	Ah and Chapman Jr. (1976)
Illinois, USA	1	An adult Beagle that whelped 6 pups, diagnosed to have *Paragonimus* eggs in feces and a cyst in lung. **Medicated with albendazole (30 mg/kg body weight, mixed in dog food for 12 days**), eggs not seen on day 8 after treatment, necropsied because of cystitis, **a dead fluke found in the pleural cavity; cysts were not detected in lungs,** eggs present in granulomatous pulmonic lesions**.**	Todd Jr. et al. (1978)
Pennsylvania, USA	1	Four-years-old mixed breed male with chronic cough, gagging, **occasional hemoptysis**, and weight loss. Diagnosis confirmed by presence of *P. kellicotti* eggs in feces, and radiography. Dog medicated with **praziquantel (5 mg/kg body weight) subcutaneously for 2 days**. Because *P. kellicotti* eggs were still present, **the dog was medicated with praziquantel (25 mg/kg body weight) orally, 3 times a day for 2 days. Treatment was successful; the dog gained weight and parasite eggs were no longer detectable 2 months later.**	Kirkpatrick and Shelly (1985)
Missouri, USA	2	Paragonimiasis diagnosed in **2 adult University Research Colony dogs, based on radiographs and *P. kellicotti* eggs in tracheal washings. Both dogs treated successfully with praziquantel (25 mg/kg/body weight, thrice daily, total doses 2.7** **g and 3.0 g). Eggs were detected in feces of 1 dog for 14 days post treatment in 1 dog and day 5 in the other dog.**	Kern (1991)
Wisconsin, USA	2	**A 3-years-old beagle female found dead, 2 other beagles in the same household died within3 weeks with clinical signs of coughing**. Necropsy of the index case revealed **3–5 cm diameter nodules in lungs**. *P. kellicotti* adult flukes were 7.5**–16 mm long** and 4–8 mm thick**. The dogs had consumed crayfish**. Second dog, 5-years-old Airedale spayed female had intermittent cough. Paragonimiasis was diagnosed based on radiography and presence of *P. kellicotti* eggs in feces. **Dog was treated successfully with fenbendazole (50 mg/ Kg body weight) orally once a day for 10 days, eggs not seen at the termination of treatment, cough had subsided.**	Madden et al. (1999)

The youngest infected cat was 3.5-months-old and the youngest dog was 3-months old (Table 6). Most cases were solitary infections, except three beagles in one household dog died of respiratory distress within three weeks; *P. kellicotti* infection was confirmed in one of these dogs examined at necropsy (Madden et al., 1999). The owner reported that the dogs had eaten crayfish the previous summer (Madden et al., 1999).

Stress might have aggravated or initiated onset of clinical signs in some cases (Table 6). A cat became dyspneic four days after orchiectomy (Rochat et al., 1990a) and a dog became dyspneic four days after ovariohysterectomy (Comfort and Axelson, 1962).

In all but one case, infections were confined to lungs. The exception was a mongrel dog from Atlanta, Georgia. This dog was also the most heavily infected; all lobes of the lungs had nodules that contained a total of 59 flukes, up to four flukes per nodule (Ah and Chapman Jr., 1976). This dog had extrapulmonary lesions with *Paragonimus* eggs. *Paragonimus* eggs were found in granulomatous lesions in scrotum, spermatic cord, liver, and mediastinal lymph nodes (Ah and Chapman Jr., 1976). It is uncertain if the dog had *P. kellicotti* or other species of *Paragonimus* (Table 6).

Before the development of albendazole and fenbendazole therapy in 1970's (Dubey et al., 1977b; Dubey et al., 1979b), paragonimiasis in cats and dogs was treated with lobectomy (Bisgard and Lewis, 1964; Rendano Jr., 1974; Pechman, 1976) or bithionol (Greve, 1969; Macy and Todd Jr., 1975; Pechman, 1976) and a variety of other drugs (Rochat et al., 1990b). Subsequently cats and dogs were treated with albendazole (Todd Jr. et al., 1978; Hoskins et al., 1981; Johnson et al., 1981), fenbendazole (Rochat et al., 1990b; Madden et al., 1999; Peregrine et al., 2014) or praziquantel (Kirkpatrick and Shelly, 1985; Kern, 1991; Table 6). In most cases responses to therapy could be followed by necropsy examination. Whether there are regional parasite strain differences with respect to chemotherapeutic is a challenging question (Peregrine et al., 2014; see Table 6). For example, albendazole therapy was successful in 8 of 10 cats in Louisiana, USA (Hoskins et al., 1981). In the other two cats, *Paragonimus* eggs or flukes were still present despite three-week therapy (Hoskins et al., 1981). There are individual variations in efficacy of chemotherapy. For example, of two dogs treated with praziquantel, eggs were not seen five days after therapy in one dog, but eggs were still present on the 14th day after treatment in another dog (Kern, 1991). Although triclabendazole and other drugs have been used to treat humans and animals infected with *P. westeramani* in other countries, the present discussion was limited to *P. kellicotti* infections in the USA and Canada (Liu et al., 1999; Keiser et al., 2005; Richter, 2022.

## 6 Human infections with *P. kellicotti*

*Paragonimus kellicotti* infections in humans in USA and Canada have been reviewed (Procop, 2000; Fischer and Weil, 2015; Blair, 2022). One report from Canada described paragonimiasis in four patients that were immigrant to Canada (Béland et al., 1969). I have not discussed this report further because of uncertainty of the species of *Paragonimus* involved. Following are the salient features of human paragonimiasis in USA.

### 6.1 Geographic distribution

Although autochthonous *Paragonimus* infections were reported from Oklahoma (Procop, 2000), Michigan (DeFrain and Hooker, 2002), Colorado (Boé and Schwarz, 2007), and Nebraska (Madariaga et al., 2007), 15 of the 20 reports of were from Missouri (Lane et al., 2009, Lane et al., 2012; Johannesen and Nguyen, 2016; Horn et al., 2016; Bahr et al., 2017). It is most likely that paragonimiasis was recognized more often in Missouri because of the interest of physicians and researchers at the Washington University, St. Louis, Missouri.

### 6.2 Risk factors

The ingestion of infected crayfish is the only confirmed mode of transmission of *P. kellicotti* and definitive data are available only from patients who went on boat trips in Missouri and ate crayfish; patients were mostly intoxicated by drinking alcohol and had recalled eating raw crayfish. Although an incubation period of two weeks is mentioned (Bahr et al., 2017; Pachucki et al., 1984), three weeks is the minimum time when symptoms of paragonimiasis were observed (Lane et al., 2009; Lane et al., 2012). This three-week period coincides with the time when *P. kellicotti* successfully enters lung parenchyma (in experimentally infected cats and dogs).

### 6.3 Symptoms

Most common localized symptoms were related to pulmonary infections (cough, chest pain, dyspnea, hemoptysis) (Henry et al., 2012;Fischer and Weil, 2015; Coogle et al., 2022). Generalized symptoms (fever, malaise, myalgia, sweats, sore throat, arthralgia, and headache) were common to toxoplasmosis (Dubey, 2022). Dermal lesions were reported twice, brain lesions in three cases, and neurologic deficits in three cases (summarized by Coogle et al., 2022).

Because these extrapulmonary signs were not seen in dogs or cats (experimentally or naturally) infected with *P. kellicotti*, these human cases are summarized in here (Table 7). Pathogenesis of occurrence of dermal and neural lesions noted in these patients is unexplained because *P. kellicotti* stages were not found and the diagnosis was primarily based on serological tests and response to praziquantel. Other salient features of human cases were:

1. Thoracoscopy and thoracostomy and placement of a tube to drain fluid from of a 35-year-old man with dyspnea revealed 800 ml of fluid, and severe pleuritis with masses of *Paragonimus* eggs in granulomatous tissue (Castilla et al., 2003).
2. Empyema (pus in pleural cavity) in an 18-year-old man was treated with praziquantel and thoracotomy (DeFrain and Hooker, 2002). There was severe pleuritis and 800 ml of fluid was drained from pleural cavity. This patient also had diarrhea three days after eating raw crayfish.
3. A 21-year-old man coughed blood every morning for six months (Procop et al., 2000). He had coughing episodes lasting upto 1h. Microscopic examination of bronchial fluid revealed *Paragonimus* eggs*.*
4. Most reports of paragonimiasis were in young adults, none of them were fatal, because cases were diagnosed and treated. However, a 71-year-old man died probably due to bacterial infection (Madariaga et al., 2007). Pleural fluid the time of admission revealed remnants of a degenerated fluke, considered *P. kellicotti* because patients had not travelled outside USA (Madariaga et al., 2007).
5. One patient had cholecystectomy because of right upper quadrant pain but *P. kellicotti* was not found in gallbladder. Authors suggested that migration of fluke through diaphragm might have caused the pain (Lane et al., 2012).

**Table 7:** Extrapulmonary lesions in cases of human paragonimiasis in USA.

Lesion/condition^a^	Diagnosis basis	Praziquantel therapy	Notes	Reference
26-year-old patient developed a 5-mm **nodule on the lower left lip** that enlarged to 15-mm and migrated to her left cheek; biopsy revealed eosinophilic infiltrate	ELISA (Parasitic Disease Consultants)	Successful. Cheek nodule resolved after 7-day treatment	Crayfish ingested 2 weeks ago	Lane et al. (2009)
32-year-old patient had headaches and blurred vision. The patient reported **having a migratory nodule** on the left fourth distal interphalangeal joint	ELISA (CDC)	Successful. Pulmonary, cerebral, and cutaneous condition resolved after 3-day treatment	Crayfish ingested 3 weeks ago	Lane et al. (2009)
32-year-old man with severe headaches, **blurred vision**	Immunoblot (CDC)	Serum eosinophilia resolved; headaches improved	Denied eating raw crayfish	Bahr et al. (2017)
18-year-old man, ataxia, severe headaches, **hydrocephalus**	Western blot (Washington University)	Successful, 4 days treatment	Ate raw crayfish	Bahr et al. (2017)

a Salient features in bold.

### 6.4 Diagnosis

Diagnosis of human paragonimiasis in the USA and Canada has entailed detection of eggs, clinical symptoms, medical history, and antibody detection. Surgical intervention was performed in one patient. Diagnosis of human paragonimiasis presents several problems because it is rare, symptoms are vague, and five weeks or more are needed for worms to mature and produce eggs (Fischer and Weil, 2015). Chronic cough and hemoptysis simulate other diseases such as tuberculosis and fungal infections. Blood eosinophilia is a consistent feature of paragonimiasis (Procop, 2000; Horn et al., 2016). Egg excretion is erratic and fecal examinations are rarely performed in patients with pulmonary ailments. The sensitivity of detection of eggs in feces or sputum is also low. Serological and molecular methods have been employed in few patients. To alleviate concerns of cross reactivity with other *Paragonimus* species, antigens from in vivo (in hamsters or gerbils) cultivated *P. kellicotti* are being exploited with success (Fischer et al., 2011; Fischer et al., 2013; McNulty et al., 2014; Curtis et al., 2021; Di Maggio et al., 2022). The genome of *P. kellicotti* is ∼700 megabases large with about 12,800 genes (Rosa et al., 2020). This facilitated proteomics studies that can improve diagnostics and provide targets for novel treatments for paragonimiasis (Di Maggio et al., 2022). The official serological reference laboratory for paragonimiasis in the USA is the CDC in Atlanta, Georgia that performs IgG detection using several diagnostic *Paragonimus* antigens (Slemenda et al., 1988; Fischer et al., 2013). Studies of genomics and transcriptomics of *Paragonimus* species, including *P. kellicotti* provide basis for improved diagnostics and therapy (Rosa et al., 2020; Di Maggio et al., 2022).

### 6.5 Treatment

Although there are many drugs available to treat paragonimiasis in humans, praziquantel is the most commonly used in the USA (Calvopiña et al., 1998; Keiser et al., 2005; Procop, 2000; Fischer and Weil, 2015; Richter, 2022). Of 21 cases of *P. kellicotti* infections in humans in USA, 20 were treated with praziquantel (reviewed in Coogle et al., 2022). The usual dosage is 25 mg/kg/body weight, three times daily for 2–3 days (Coogle et al., 2022).

## 7 Conclusions and perspective

Here, I have reviewed biology of *P. kellicotti* in animals and humans in USA and Canada. Among many species of wildlife, the mink is likely the most important reservoirs of *P. kellicotti* infection. The infective stage of the parasite, metacercariae are produced year around and infections are common in crayfish. Thus, people or animals are likely to become infected by ingesting raw crayfish, as has happened in people who were intoxicated with alcohol while on boat trips in fresh waters. Most symptoms are related to pulmonary infection, and death can occur due to pneumothorax resulting from cyst rupture. The pathogenesis of extrapulmonary symptoms in paragonimiasis in humans remains unclear, because these symptoms/signs have not been documented in cats or dogs. Sensitive and simple diagnostic methods, now in development, should aid research and clinical management.

### 7.1 Specimens deposited

The specimens were deposited in the United States National Parasite Collection in the Division of Invertebrate Zoology and National Museum of Natural History, Smithsonian Institution, Museum Support Center, MRC 534, 4210 Silver Hill Road, Suitland, Maryland 20746, USA (Table 8).

**Table 8:** Details of *Paragonimus kellicotti* specimens from experimentally infected cats or dogs deposited in the Smithsonian Museum.

**ID**	**Day p.i.**	**Host**	**Tissue**	**Stain**	**Fig. no.**	**Museum no.**
**1** (98B)	7	Cat	Whole fluke	Carmine	3A	USNM 1675982
**2** (33)	14	Cat	Whole fluke	Carmine	3B	USNM 1675983
**3** (66)	21	Cat	Whole fluke	Carmine	3C	USNM 1675984
**4** (Aa)	29	Cat	Whole fluke	Carmine	3D	USNM 1675985
**5** (Bb)	34	Cat	Whole fluke	Carmine	3E	USNM 1675986
**6** (Y675-5)	10	Cat	Histological section of lung, *P. kellicotti* entering pleura	HE	5A	USNM 1675987
**7** (Y686-2)	14	Cat	Histological section of lung, *P. kellicotti* below pleura	HE	5B	USNM 1675988
**8** (Y686-6)	14	Cat	Histological section of lung, *P. kellicotti* pair in lung cavity	HE	5C	USNM 1675989
**9** (X2050-1)	21	Cat	Histological section of lung, inflammatory host cells, and hemorrhage around *P. kellicotti*	HE	5D, E	USNM 1675990
**10** (Y4133-2)	29	Cat	Histological section of lung, *P. kellicotti* pair in early cyst	HE	6A	USNM 1675991
**11** (X2040-1)	39	Cat	Histological section of lung, *P. kellicotti* pair in cyst communicating with bronchus	HE	6B	USNM 1675992
**12** (X2696-2)	70	Cat	Histological section of lung, *P. kellicotti* oral sucker feeding/attached to cyst wall	HE	7A	USNM 1675993
**13** (T5033-9)	70	Dog	Histological section of lung showing *P. kellicotti* eggs among degenerating host tissue	HE	7B	USNM 1675994
**14** (X2096-4)	122	Cat	Histological section of lung, 21 days after treatment with albendazole	HE	9A	USNM 1675995
**15** (X2096-2)	122	Cat	Histological section of lung, 21 days after treatment with albendazole	HE	9B	USNM 1675996
**16** (T5033-9)	70	Dog	Lung, paraffin block, day 70 p.i.	None	None	USNM 1675996

## Declaration of Competing Interest

The authors declare that they have no known competing financial interests or personal relationships that could have appeared to influence the work reported in this paper.
